# Supplementary material for: Unveiling the Intimate Mechanism of the Crocin Antioxidant Properties by Radiolytic Analysis and Molecular Simulations
Source: Antioxidants (Basel). 2023 Jun 1;12(6):1202. doi: 10.3390/antiox12061202 (PMC10295680; doi:10.3390/antiox12061202)
Supplement: Supplementary file 1 [file antioxidants-12-01202-s001.zip › antioxidants-2394878-supplementary.pdf]

Supplementary Information

Unveiling the Intimate Mechanism of the Crocin Antioxidant Properties by Radiolytic Analysis and Molecular Simulations

Sarah Al Gharib, Pierre Archirel *, Daniel Adjei, Jacqueline Belloni and Mehran Mostafavi *

Institut de Chimie Physique, Université Paris-Saclay, UMR8000 CNRS, Rue Michel Magat, F-91405 Orsay, France; sarah.al-gharib@universite-paris-saclay.fr (S.A.G.); daniel.adjei@universite-paris-saclay.fr (D.A.); jacqueline.belloni@universite-paris-saclay.fr (J.B.)

* Correspondence: pierre.archirel@universite-paris-saclay.fr (P.A.); mehran.mostafavi@universite-paris-saclay.fr (M.M.)

3. Radiolytic Oxidation Results

3.1. Solubility of Crocin

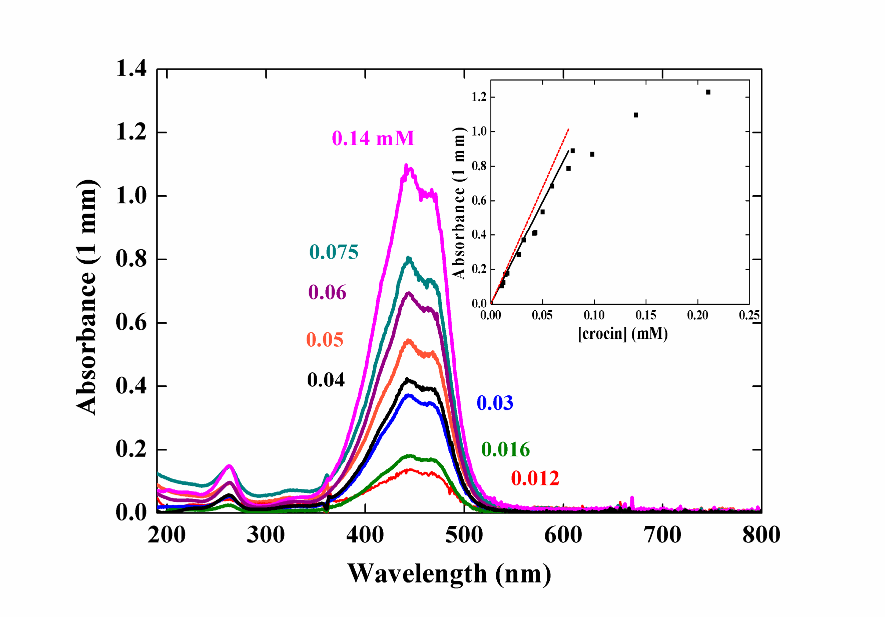

Figure S1. Optical absorption spectra of crocin at various concentrations (calculated from the amount in g mL^{-1}). **Inset:** Dependence of the absorbance at 441 nm on the crocin concentration. Dotted line in red: Absorbances calculated with the literature average value of the molar absorption coefficient ε_{441}(croc) = 1.35 × 10^{5} M^{-1} cm^{-1}.

The Figure S1 presents the absorbance of aqueous crocin solutions at various concentrations calculated from the crystals weight. In the Inset, the absorbance increases first linearly with this concentration, but progressively reaches an asymptotic value. Clearly, the slope then decreases above 0.10 mM, meaning that some changes occur in the solution, possibly by association of molecules. Finally, at the plateau ([croc] > 0.15 mM), the crocin reaches a solubility upper limit. The initial slope (in black)

corresponds to a molar absorption coefficient ε_{441}(croc) = 1.18 × 10^5 M^{-1} cm^{-1}. It is lower than the dotted line (in red) corresponding to the average value ε_{4441}(croc) = 1.35 × 10^5 M^{-1} cm^{-1} of the molar absorption coefficients of the literatur $\varepsilon\varepsilon_{4441}$(croc) = 1.369 × 10^5 M^{-1} cm^{-1} [3],and ε_{4441}(croc) = 1.335 × 10^5 M^{-1} cm^{-1}-. The solution thus contains some impurity that does not absorb around 441 nm. All the concentrations in the text are calculated with the mean value of the literature data.

3.2. Reaction of Crocin with OH$^•$ Radical within 1 μs

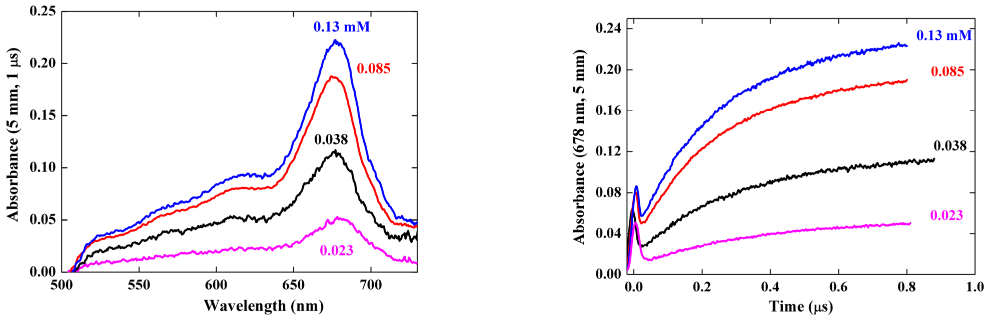

(a) (b)

Figure S2. (a) Transient difference spectra in the visible at 1 s after the pulse for various crocin concentrations. (b) Kinetics at 678 nm.

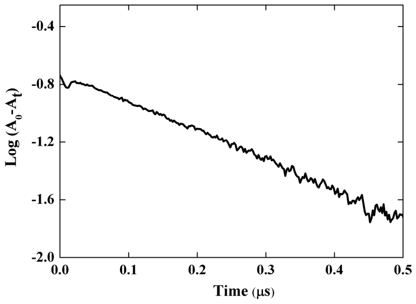

Figure S3. First order test of the absorbance increase of the radical R$^•$ = (croc(-H)$^•$) formation at 678 nm at the crocin concentration [croc] = 0.13 mM.

3.3. Reaction of the Crocin Oxidized Radical (R•) within 1 to 500 μs

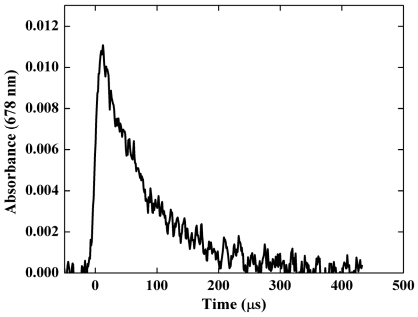

(a)

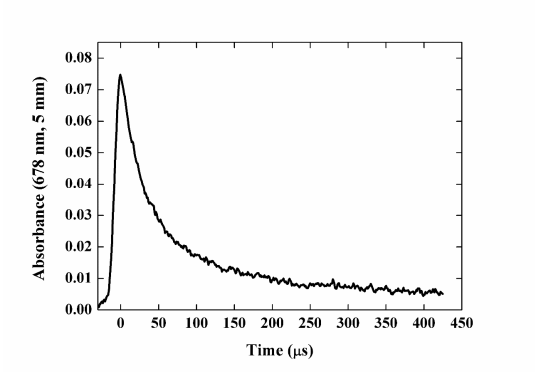

(b)

Figure S4. Kinetics at 678 nm within 500 μs for a. [croc] = 0.02 mM, b. [crocin] = 0.038 mM.

4. Thermochemistry of Me_2-Crocetin Oxidation

4.1. Oxidation Free Energies

Species	$\Delta_r G_{abs}$ (eV)	Last MC geometry
$R^{\bullet}C_a$	- 1.98	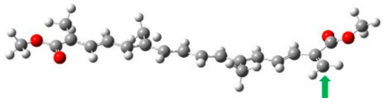
$R^{\bullet}C_3$	- 1.59	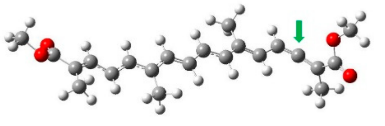
$R^{\bullet}C_4$	- 1.19	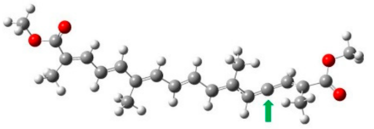
$R^{\bullet}C_5$	- 1.54	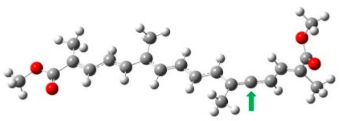
$R^{\bullet}C_b$	- 1.94	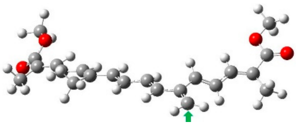

**R•C7**	- 1.49	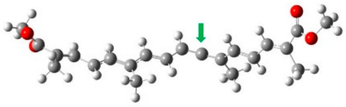
**R•C8**	- 1.43	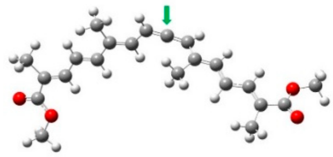
**Cation**	- 0.69	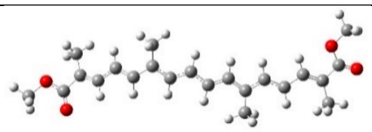

Figure S5. Possible oxidation products of Me_2-crocetin: values of the free energy for H abstraction and ionization, and typical configuration of the MC simulation. Oxidized carbon atoms are emphasized with a green arrow.

4.2. Transition States

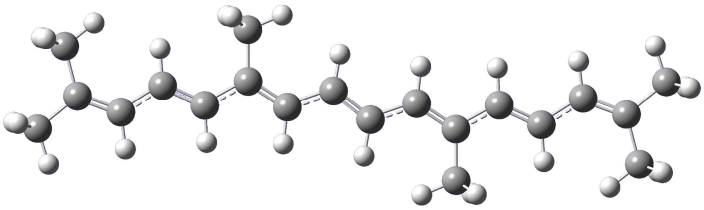

Figure S6. Model 2 (hydrocarbon) for the evaluation of transition structures.

We first calculated the association free energy of the OH• radical with Me_2-crocetin. We used the large *apvdz* basis and investigated the following possibilities: (i) choice of the functional: B3LYP or cam-B3LYP, which is better suited to conjugated systems, (ii) inclusion of empirical dispersion and (iii) correction of entropies with the Wertz formula, according to Formula (5). The results are given in Table S1. The most reliable value of the association free energy is -0.62 eV, with cam-B3LYP, empirical dispersion and entropy correction. It is striking that the same result is obtained with B3LYP and no dispersion. We will use this method for the calculation of transition states, because its convergence is much easier. Mulliken spin density shows that the complex is of the ionic type (Me_2-crocetine$^+$, OH^-). Table S1 also shows that the free energy value does not depend on which oxygen atom of the -$COOCH_3$ group is bound to OH^-. The molecular structure is shown in Figure S7.

Table S1. Association free energy (in eV) of the $OH^{•}$ radical with Me_2-crocetine with influence of the functional, of dispersion interactions and of entropy correction. The method is DFT / apvdz / SMD. We tested the two O atoms of the $-COOCH_3$ group.

O atom	Functional	Dispersion	Crude	Corrected
**-OCH3**	B3LYP	no	- 0.501	- 0.623
**-OCH3**	B3LYP	yes	- 0.558	- 0.687
**-OCH3**	cam-B3LYP	no	- 0.464	- 0.586
**-OCH3**	cam-B3LYP	yes	- 0.488	- 0.618
**=O**	B3LYP	no	- 0.523	- 0.625

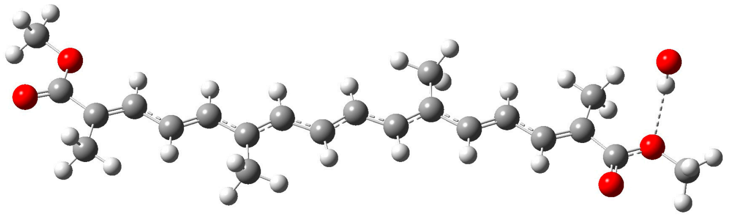

Figure S7. Structure of the (Me_2-crocetin$^{•+}$, OH^{-}) adduct.

Then we applied the same method to the transition states: (i) use of the B3LYP functional and of the SMD model, (ii) calculation of the nuclear hessian at every optimization step, and (iii) highest quality (superfine) integration grid for the DFT. The transition states have been identified with the usual criteria: (i) they have one (and only one) imaginary frequency, (ii) the movement along the corresponding eigen direction of the nuclear hessian shows H transfer between the CH group and the $OH^{•}$ radical [41] and (iii) the IRC method [38] was applied whenever possible.

We first used the IRC method [38], with incomplete success: (i) the method succeeded and confirmed the visual clue for the TS with large imaginary frequencies, such as that of carbon C_3, but (ii) the IRC method could not manage the TS with several small imaginary frequencies, such as that for carbons C_a and C_b. In this last case we performed two geometry optimizations from two starting points in the close vicinity of the TS, taken along the eigen direction of the nuclear hessian [41]. This optimization yielded both expected stationary points, (Me_2-crocetine, $OH^{•}$) on one side, and (Me_2-crocetine (-H)$^{•}$, H_2O) on the other side, therefore mimicking the IRC method. Typical TS structures are given in Figure S7, with structural parameters and partial charges. We also compared the values of the barrier heights of the -C_3H and the -C_7H groups with the two bases. This enabled us to obtain an approximation of the barrier height in Me_2-crocetin. The results are given in Table S2.

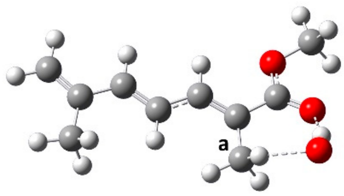	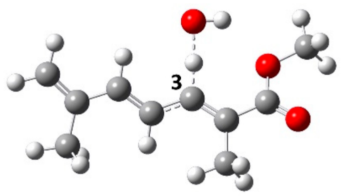	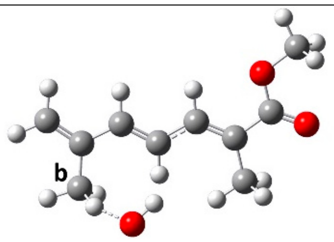
C_aH : 1.094, 1.098, 1.112*<br>OH : 0.991, 2.031*<br>q(OH) = - 0.10	C_3H : 1.999*<br>OH : 0.974, 1.398*<br>q(OH) = - 0.32	C_bH : 1.096, 1.099, 1.114*<br>OH : 0.976, 1.999*<br>q(OH) = - 0.19

Figure S8. Three typical transition states in Me_2-crocetine oxidation by the $OH^\bullet$ radical. The model 1 of Me_2-crocetine (Figure 9) is used. Typical CH and OH distances are given in Å. Starred values refer to the H atom engaged in the transition state. NBO charges in a. u. are also given. The method is B3LYP / aug-cc-pvdz / SMD.

Table S2. TS free energy (in eV) and imaginary frequency (in cm^{-1}, with a negative sign) for the oxidation of the C_3H and C_aH_3 groups by the OH and NO_2 radicals. We compare two gaussian bases and three systems. Model 1 refers to Figure 9, model 2 is shown in Figure S8. The bracketed numbers have been deduced.

System	Radical	Carbon	pSDD	apvdz
Model 1	$OH^\bullet$	C_3	0.28 (-1522)	0.12 (-1312)
Me_2-crocetin	$OH^\bullet$	C_3	0.23 (-1566)	(0.07)
Model 1	$OH^\bullet$	C_7	0.25 (-1632)	0.08 (-1516)
Me_2-crocetin	$OH^\bullet$	C_7	0.21 (-1624)	(0.04)
Model 2	$OH^\bullet$	C_a	- 0.57 (-1273)	-
Model 2	$NO_2^\bullet$	C_a	+ 0.27 (-1584)	-

4.3. Conformation Analysis

Table S3. Free energies *G* (eV), absorption data λ_{max} (nm) and oscillator strength *f* of the *all-trans* and *cis-trans* conformers of crocin and Me_2-crocetin, obtained by rotation about one chemical bond of the *all-trans* molecule. The bonds are defined by the numbers of the ~~two~~ atoms in Figure 1. We give the values for the first 2 transitions at the lc-ωPBE (0.244) / psDD / SMD level. Starred values *G** refer to the *apvdz* basis set.

**Crocin**			**Me_2-crocetin**			
**Bend**	**G (eV)**	**λ_{abs} (nm) / f**	**Bend**	**G (eV)**	**λ_{abs} (nm) / f**	**G* (eV)**
all-trans	0.	447 / 3.4318	***all-trans***	0.	441 / 3.2245	0.
		322 / 0.0027			316 / 0.0002	
**(6,7)**	+0.075	443 / 2.6891	**(6,7)**	+ 0.070	437 / 2.6195	+ 0.079

		324 / 0.7433			319 / 0.6318	
**(2,3)**	+0.12	448 / 3.3695	**(2,3)**	+ 0.076	443 / 3.1782	+ 0.074
		324 / 0.0520			319 / 0.0284	
**(8,8′)**	+0.15	443 / 2.4449	**(8,8′)**	+ 0.11	437 / 2.3808	+ 0.088
		322 / 1.0360			317 / 0.9124	

Table S4. Free energies *G* (eV), absorption data λ_{max} (nm) and oscillator strength *f*, and mean value of the spin operator S^2 (a. u.) of the cis-trans conformers of the radicals $R^{\bullet}C_a$ and $R^{\bullet}C_b$ of Me_2-crocetine, obtained by rotation about one and two chemical bonds of the all-trans molecule. We give the values for the first three transitions of $R^{\bullet}C_a$ and the first four transitions of $R^{\bullet}C_b$, at the lc-ωPBE (0.244) / psDD / SMD level. Blank cells ensure that the intense transitions of each species are on the same line.

$R^{\bullet}$ C_a				$R^{\bullet}$ C_b			
**Bend**	**G (eV)**	**λ_{abs} (nm) / f**	**$\langle S^2 \rangle$ (a. u.)**	**Bend**	**G (eV)**	**λ_{abs} (nm)/ f**	**$\langle S^2 \rangle$ (a. u.)**
all-trans	0.	659 / 0.0012	1.484	**(6,7)**	0.	686 / 0.0011	2.620
		566 / 0.0930	1.950			570 / 0.0283	1.326
		-	-			468 / 0.0801	2.034
		462 / 2.8131	1.086			414 / 2.1841	0.957
**(5,6)**	0.032	683 / 0.0042	1.683	***all-trans***	0.002	735 / 0.0007	2.529
		574 / 0.0761	1.790			575 / 0.0169	1.327
		-	-			478 / 0.1655	2.035
		468 / 2.2089	1.145			424 / 2.1263	1.037
**(2,3)**	0.049	647 / 0.0064	1.343	**(5,6)**	0.025	755 / 0.0004	2.693
		558 / 0.0769	2.019			567 / 0.0312	1.205
		-	-			461 / 0.0707	2.067
		462 / 2.9921	1.078			413 / 2.0818	0.951
**(2′, 3′)**	0.076	665 / 0.0001	1.456	**(5,6) (6,7)**	0.053	709 / 0.0005	2.657
		577 / 0.1099	1.943			568 / 0.0348	1.259
		-	-			463 / 0.0389	2.054
		471 / 2.7823	1.126			414 / 1.9722	0.945

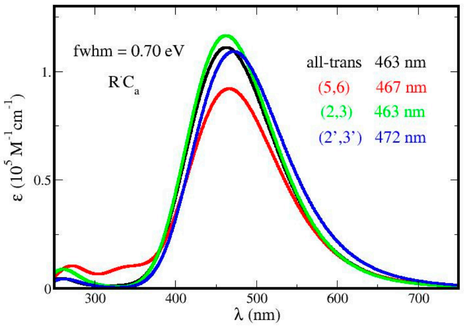

(a)

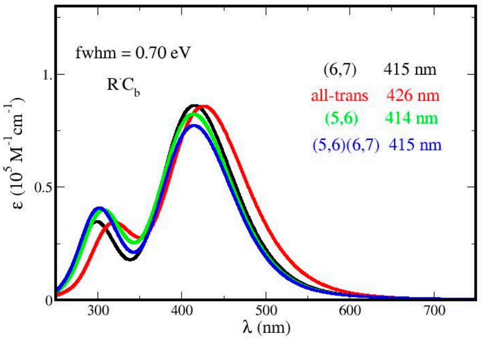

(b)

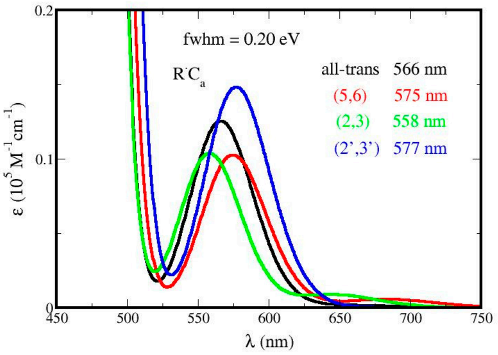

(c)

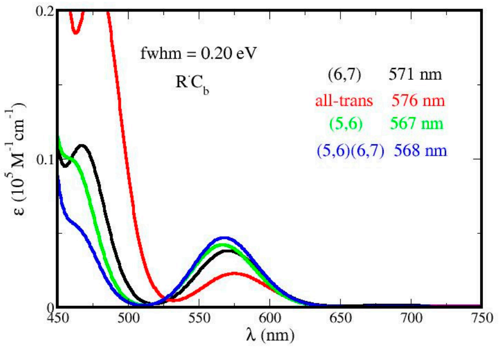

(d)

Figure S9. R•C_a (left) and R•C_b (right) radicals: absorption spectra of the lowest four conformers through broadening of the TDDFT data of the optimized structures of Table SI 4. No spin screening has been done. We show the intense bands with *fwhm* = 0.70 eV (top) and the weak, low energy bands with *fwhm* = 0.20 eV (bottom).

5. Molecular Simulations

5.0. Influence of the Simulation Parameters

The influence of the two critical parameters, the *fwhm* parameter of the convolution of TDDFT lines, and the spin screening parameter were investigated. This is done on the case of the R•C_a species, and the results are shown on Figure S10. It can be seen that:

1. Figure S10a shows that increasing the value of *fwhm* between 0.1 eV and 0.7 eV strongly modifies the value of the absorption parameter and leaves the absorption wavelength λ_{max} unaffected. The value 0.5 eV yields the order of magnitude of the observed value of ε: 10^5 M^{-1} cm^{-1}.
2. Figure S10b shows the same spectra with a focus on weak bands. It can be seen that low energy bands are visible with *fwhm* = 0.2 eV, and that these bands are concealed if larger value are used. We have here the issue that description of both the intense band and low energy, weak bands is impossible if the same value of *fwhm* is used.
3. Figure S10c shows the two spectra obtained with the values M_s = 1/2 and 3/2 of the z component of the total electronic spin. These spectra show that spin contamination in the M_s = 1/2 spectrum can be expected small in the 400 - 500 nm intense band, where the absorption of the M_s = 3/2 species is

weak, and large in the low energy zone, where the absorption of the $M_S = 3/2$ species is large. Figure S10d shows that higher energy weak bands also undergo some spin contamination.

4. Figures S10e,f show the crude spectrum, without spin screening, and the spectra obtained with different values of the spin screening. It can be seen on (e) that spin screening only reduces the absorption coefficient of the intense band, and on (f) that the value 20% clearly extracts weak bands from the crude spectrum. Using the more demanding values 10% and 5% only reduces absorption coefficients.

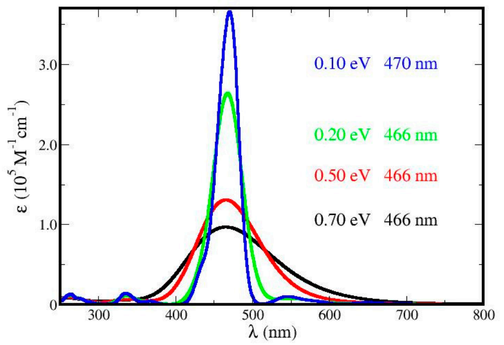

(a)

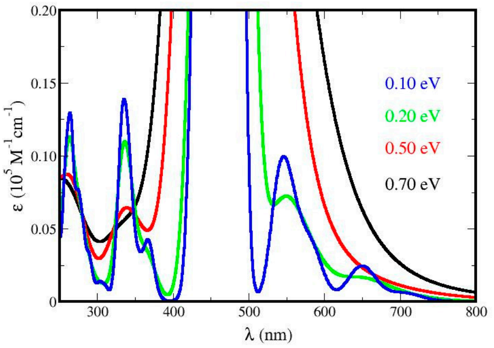

(b)

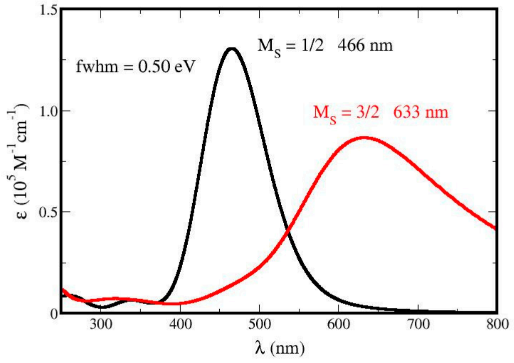

(c)

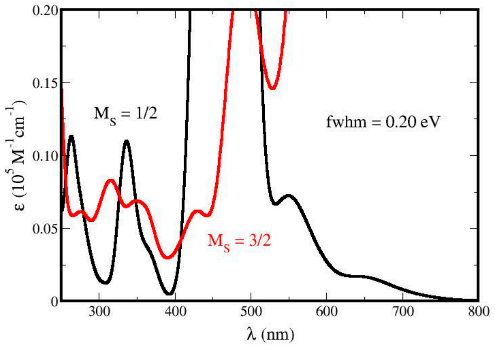

(d)

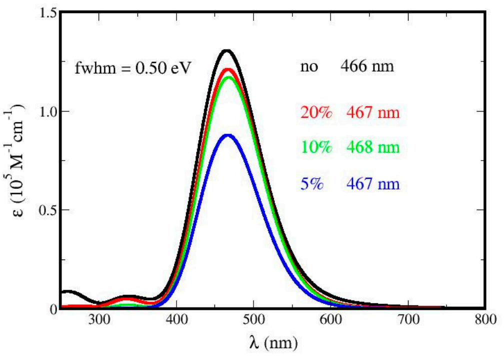

(e)

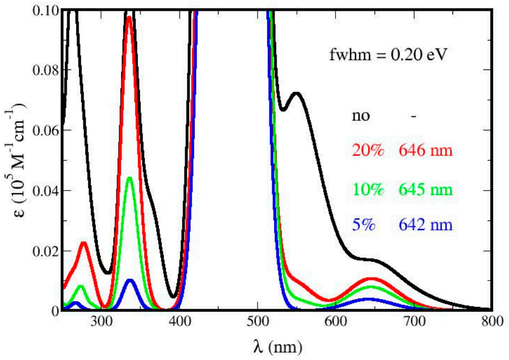

(f)

Figure S10. Simulated spectra of the R$^\bullet$C$_a$ radical of Me$_2$-crocetine. Influence of the *fwhm* parameter of the convolution on the intense band (a) and the low energy band (b). Influence of the M_S value, ½ and 3/2: (c,d). Influence of the spin screening parameter on the M_S = ½ spectru*m:* (e,f).

5.1. Absorption Spectrum of Me2-Crocetin

all-trans	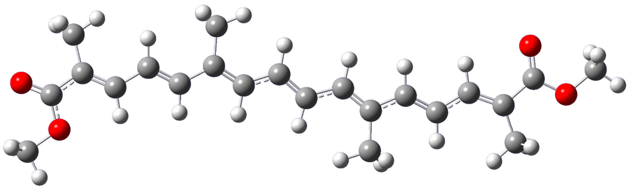
**(2,3)**	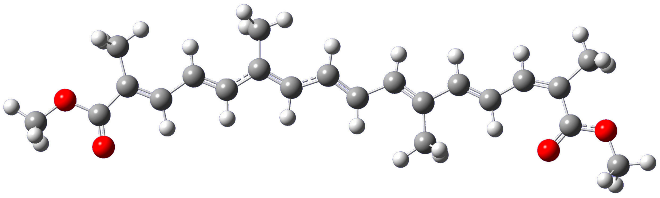
**(6,7)**	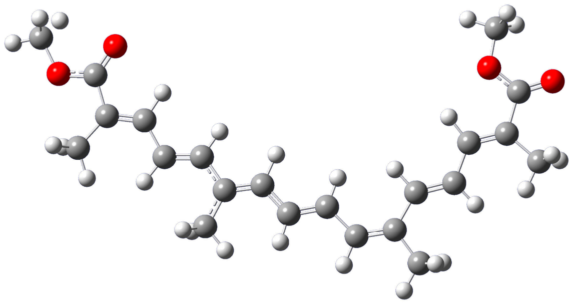
**(8,8′)**	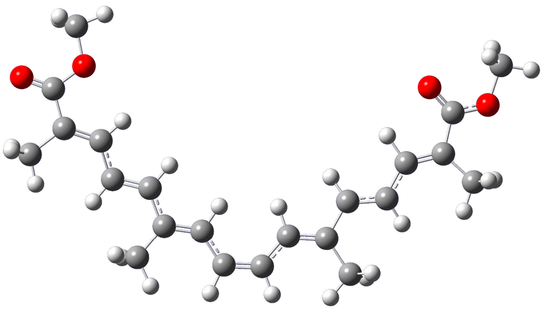

Figure S11. The four main conformations of Me2-crocetin. Last geometry of the MC simulation.

5.2. Absorption Spectra of Me2-Crocetin Radicals

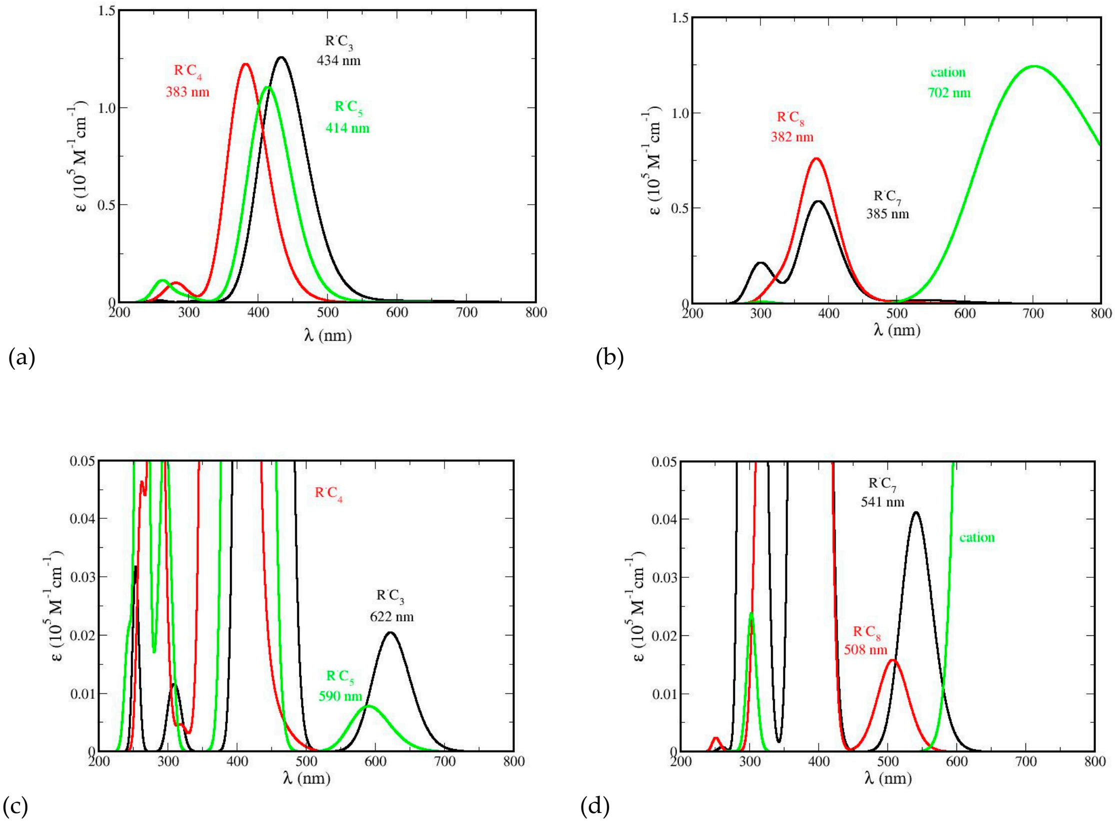

Figure S12 Simulated spectra of all the R• C species obtained by oxidation of >CH groups, and of the cation. The focus is made: (a,b). on intense bands with *fwhm* = 0.50 eV. (c,d). on low energy, weak bands, with f*whm* = 0.20 eV: Spin screening: 20%.

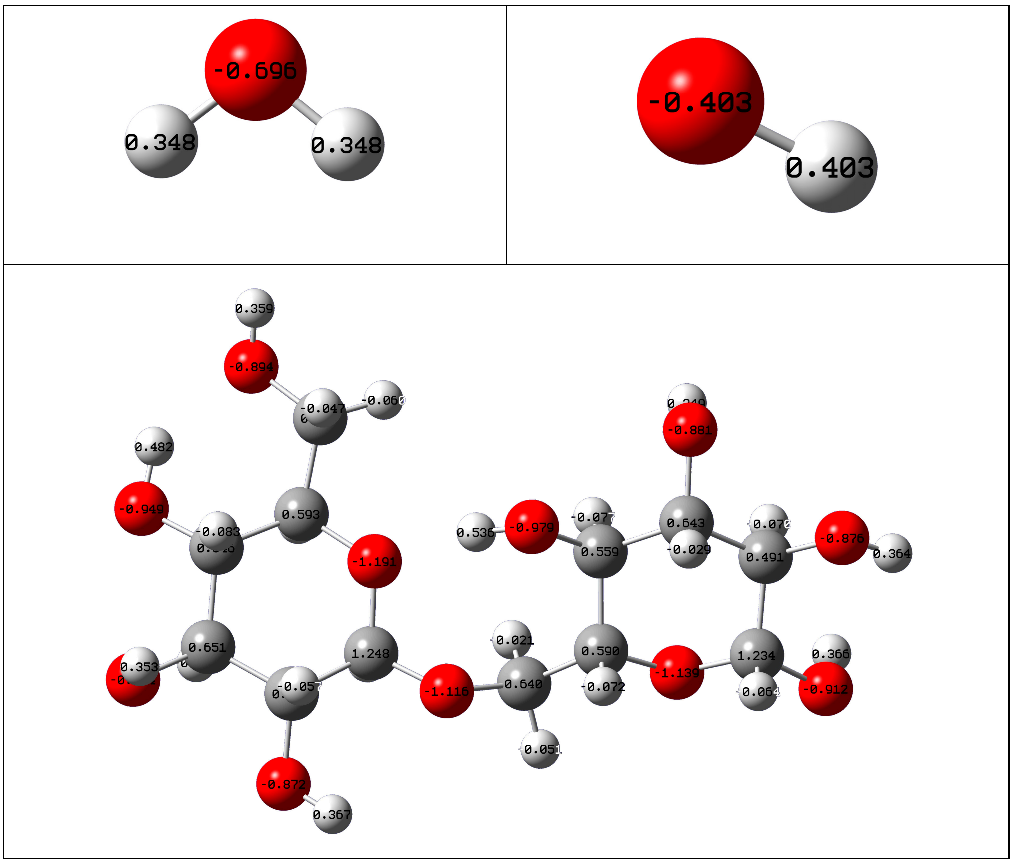

Figure S13. APT partial charges of the water molecule and OH$^{\bullet}$ radical (top) and of the gentiobiose molecule (bottom).

5.3. Absorption Spectra of Radical Dimers

**C_aC_a**	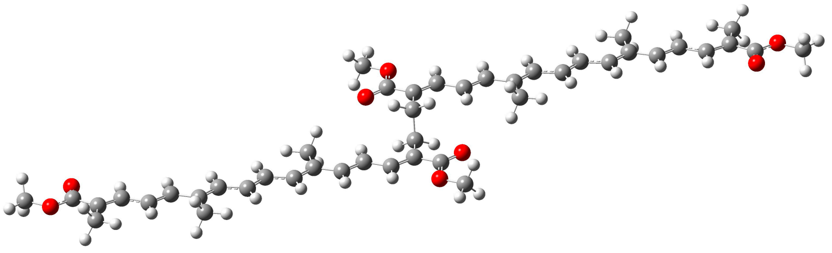
**C_aC_b**	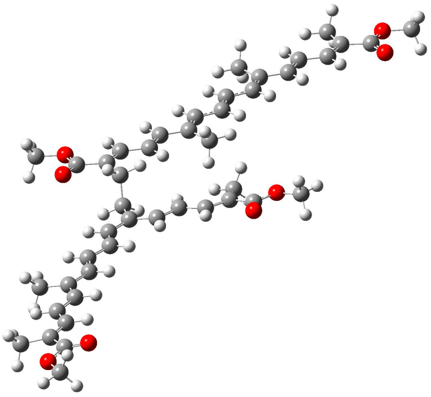
**C_bC_b**	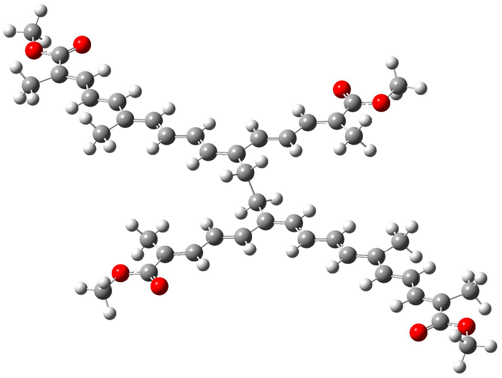

Figure S14. Optimized structures of the covalent dimers of the species $R^{\bullet}C_a$ and $R^{\bullet}C_b$.

5.4. Absorption Spectra of Disproportionation Products

**(Me2-crocetin, R•Ca)**	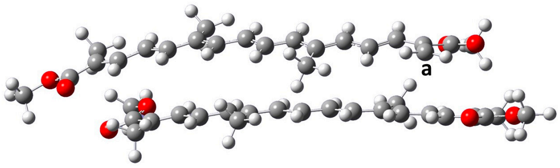
**(Me2-crocetin, R•Cb)**	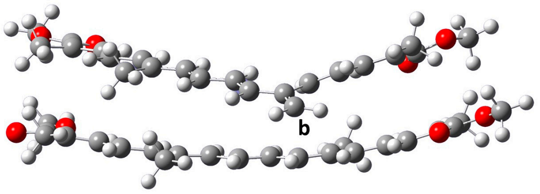
**(Glu2-crocetin, R•Ca)**	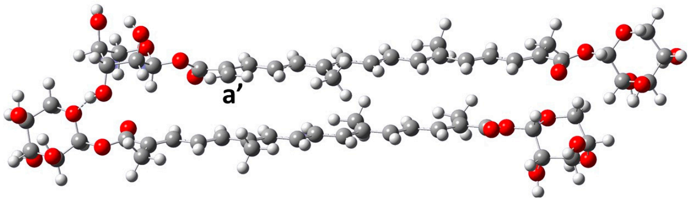

Figure S15. Chemical structures of the van der Waals complexes of Me2-crocetin and Glu2-crocetin with the corresponding radical species. The oxidized carbon atom is emphasized.

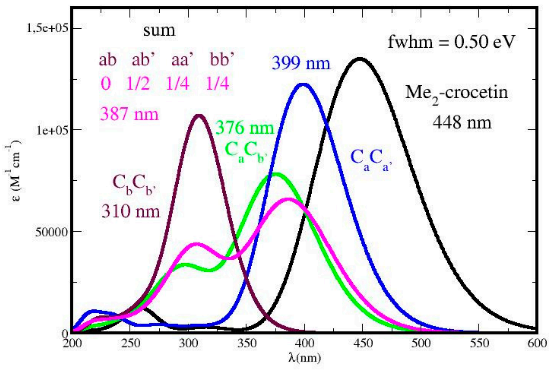

Figure S16. Absorption spectra of the primary species, before cyclisation, resulting from the disproportionation, and of one of their combinations.

	Primary species	Cyclic species
C_aC_b	not simulated	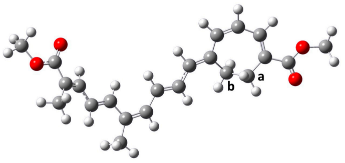
$C_aC_{b'}$	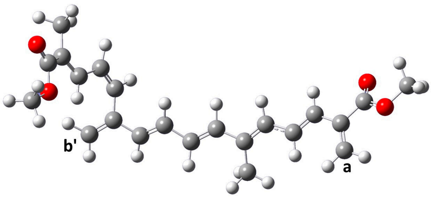	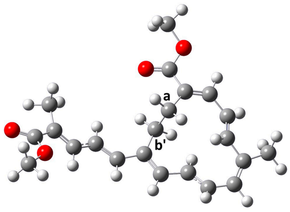
$C_aC_{a'}$	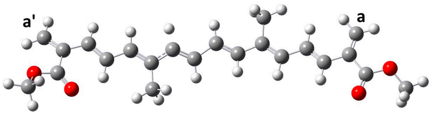	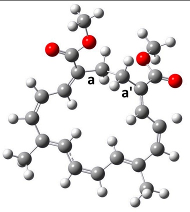
$C_bC_{b'}$	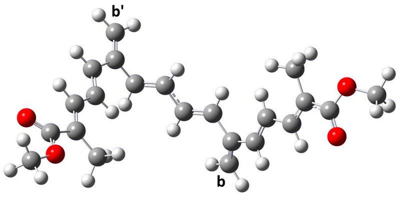	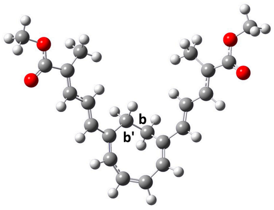

Figure S17. Products of the disproportionation of the $R^{\bullet}C_a$ and $R^{\bullet}C_b$ species (last geometry of the simulations). Left: of the primary species before cyclisation. Right: of the cyclic species. The oxidized carbon atoms are emphasized.
